# Efficacy of Eltrombopag in Children with Post-Stem Cell Transplant Prolonged Isolated Thrombocytopenia

**DOI:** 10.3390/hematolrep14030033

**Published:** 2022-08-01

**Authors:** Megumi Matsumoto, Kazuki Terada, Taichiro Tsuchimochi, Satoko Takahashi, Yasushi Noguchi, Shunji Igarashi

**Affiliations:** 1 Department of Pediatrics, Japanese Red Cross Narita Hospital, Narita 286-8523, Japan; megumi.matsumoto.mm@gmail.com (M.M.); k2095110@kadai.jp (T.T.); satoko_y0115@ga2.so-net.ne.jp (S.T.); nogunari@naritasekijyuji.jp (Y.N.); shun2@cd5.so-net.ne.jp (S.I.); 2 Department of Pediatrics and Adolescent Medicine, Juntendo University Graduate School of Medicine, Tokyo 113-8421, Japan

**Keywords:** eltrombopag, thrombopoietin receptor agonist, prolonged isolated thrombocytopenia, allogeneic stem cell transplantation

## Abstract

Prolonged isolated thrombocytopenia (PIT) is a complication following allogeneic hematopoietic cell transplantation that results in prolonged transfusion dependence. Recently, the efficacy of a thrombopoietin receptor agonist (eltrombopag) against PIT has been reported in adults; however, there are few reports in children. A 4-year-old male pediatric patient diagnosed with congenital pure red cell aplasia underwent allogeneic hematopoietic cell transplantation. Neutrophil engraftment was observed on post-transplant Day 26; however, platelet counts remained <10 × 10^9^/L. Transfusions were required 1–2 times a week for at least 4 months. On post-transplant Day 124, oral eltrombopag (up to 2.4 mg/kg/day) was initiated. Thereafter, the platelet counts were maintained at ≥10 × 10^9^/L, and the patient became transfusion independent. At 2 years and 6 months after the oral administration, no chromosomal abnormalities, thromboembolism, or myelofibrosis was observed. Thus, eltrombopag can be a potential treatment option for pediatric PIT.

## 1. Introduction

Prolonged isolated thrombocytopenia (PIT) is defined as a condition in which platelet counts do not exceed 20 × 10^9^/L at 60 days after hematopoietic cell transplantation or when the patient remains transfusion dependent despite achieving neutrophil and reticulocyte engraftment after the procedure; PIT is a complication that occurs in 5–30% of patients undergoing allogeneic hematopoietic cell transplantation [1]. Standard therapies for PIT have not yet been established; currently, platelet transfusions are recommended to prevent serious bleeding [2]. However, frequent long-term transfusions adversely affect safety and quality of life [3]. Particularly in children, the course after transplantation is long, and frequent blood transfusions can become a serious issue [4]. In adults, the efficacy of eltrombopag, a 2nd generation thrombopoietin receptor agonist (TPO-RA), against PIT has been reported as a curative alternative to blood transfusions. In children, eltrombopag has been shown to be effective against idiopathic thrombocytopenic purpura (ITP) and aplastic anemia (AA) [5,6]; however, few studies have reported the use of eltrombopag for PIT. Here, we report our experience with a pediatric patient with congenital pure red cell aplasia (Diamond–Blackfan Anemia, DBA) who was administered eltrombopag for the onset of PIT after allogeneic hematopoietic cell transplantation. We found that long-term administration of eltrombopag was effective and safe to a certain extent.

## 2. Case Report

The patient was a 4-year 9-month-old male who presented with symptoms of pale complexion and respiratory distress at the age of 1 month and was urgently admitted to a hospital. Peripheral blood examination showed macrocytic anemia and decreased reticulocytes. Bone marrow examination showed normocellular marrow with marked depletion of erythroid precursors and base substitution of ribosomal protein S19 (RPS 19) gene, which led to a diagnosis of DBA. The patient was treated with prednisolone 2 mg/kg/day at the age of 1 year and 1 month and with cyclosporine 7 mg/kg/day two months later. At the age of 2 years and 1 month, treatment with prednisolone 2 mg/kg/day was re-initiated; however, the patient did not respond to any of these treatments. The patient continued requiring red blood cell transfusions once every 4 weeks and was admitted for allogeneic hematopoietic cell transplantation. A bone marrow examination on admission showed a marked decrease in the number of erythroid cells (0.0/μL). Platelet and megakaryocyte counts were maintained (nucleated cells: 4.0 × 10^4^/μL, megakaryocytes: 50/μL).

**Figure 1:**
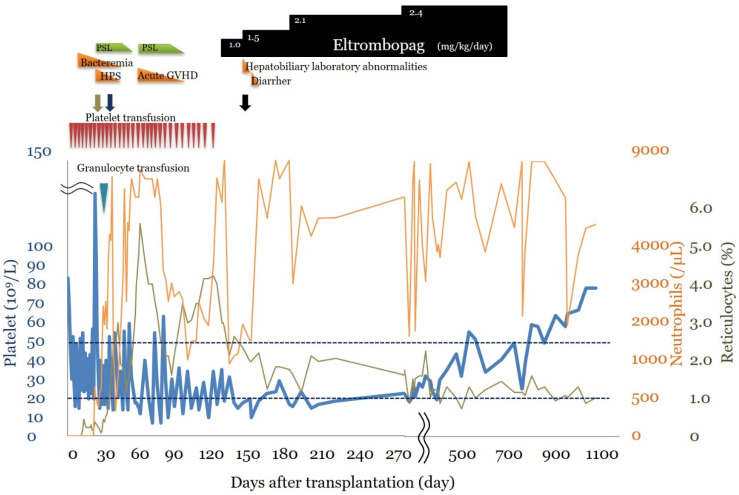
Clinical course. The clinical course after stem cell transplantation and multiple platelet transfusions. Detailed descriptions are provided in the case report. HPS: hemophagocytic syndrome, GVHD: Graft-versus-host disease. Red triangle: platelet transfusion, blue triangle: Granulocyte transfusion. Green arrow: graft survival, purple arrow: reticulocytes >1.0%, black arrow: discharge.

Clinical course (Figure 1): At the age of 4 years and 9 months, 2 doses of busulfan (1.2 mg/kg/dose from preprocedural Day 8 to Day 7), 4 doses of fludarabine (25 mg/kg/dose from preprocedural Day 5 to Day 2), 4 doses of cyclophosphamide (750 mg/kg/dose from preprocedural Day 5 to Day 2), 4 doses of antihuman thymocyte rabbit immunoglobulin (1.25 mg/kg/dose from preprocedural Day 5 to Day 2), and 3 Gy of thoracoabdominal irradiation were administered as pretreatment, and bone marrow transplantation from an unrelated complete human leukocyte antigen match donor was performed. The number of nucleated cells in the donor bone marrow was 2.4 × 10^8^/kg, and the number of CD34+ cells administered was 5.7 × 10^5^/kg. Methotrexate and FK 506 were used as graft-versus-host disease (GVHD) prophylaxis. On post-transplant Day +10, the patient developed bacteremia due to *Staphylococcus epidermidis* and was treated with meropenem and teicoplanin. On post-transplant Day +18, as the patient’s neutrophil count had not recovered, bone marrow examination was performed, which revealed hemophagocytosis; thus, prednisolone 2 mg/kg/day was administered. On post-transplant Day +21, a granulocyte transfusion (7.2 × 10^8^/kg) from the patient’s father was performed to control bacteremia, which reduced the infection. Neutrophil and reticulocyte engraftments were confirmed on Day +26 and Day +33 post-transplant, respectively, and prednisolone was tapered by Day +41 post-transplant. On post-transplant Day +48, a skin eruption was noted, and skin biopsy led to the diagnosis of acute GVHD. From post-transplant Day +56 onward, prednisolone 2.0 mg/kg/day was administered for acute GVHD, which was tapered by post-transplant Day +83. The severity of acute GVHD was ultimately Skin Stage II, Grade I. A spontaneous increase in the platelet count could not be confirmed, and platelet transfusions were required 1–2 times a week. On post-transplant Day +82, bone marrow examination showed decreased megakaryocyte counts (6/μL, nucleated cells: 15 × 10^4^/μL), immature megakaryocytes, and no other abnormal findings. A bone marrow examination on post-transplant Day +116 showed similar results (megakaryocyte count: 13/μL, nucleated cells: 10 × 10^4^/μL). There were no findings suggesting reactivation based on Epstein–Barr virus or cytomegalovirus quantification or bacterial or fungal infection. On post-transplant Day +97, a Coombs test was performed, which was negative. No abnormalities were noted in the coagulatory system. Even after post-transplant Day +120, the platelet count did not exceed 20 × 10^9^/L and transfusion dependence continued; thus, a diagnosis of PIT was established. On post-transplant Day +124, oral eltrombopag 1.0 mg/kg/day was initiated, which was increased to a dose of 1.5 mg/kg/day on post-transplant Day +137. From post-transplant Day +144 onward, the platelet count was maintained at ≥1.0 × 10^4^/μL and blood transfusions were no longer necessary, and the patient was discharged on post-transplant Day +148. On post-transplant Day +154, the platelet count met the 20 × 10^9^/L criterion. Thereafter, transfusions were no longer required; however, to maintain platelet counts within the range of 10–30 × 10^9^/L, eltrombopag was gradually increased up to a dose of 2.4 mg/kg/day until post-transplant Day +273. From post-transplant Day +294 onward, the platelet count was maintained at >20 × 10^9^/L and subsequently at ≥50 × 10^9^/L; thus, oral eltrombopag was discontinued 1 year and 10 months after its initiation, with platelet counts stabilizing thereafter. White blood cell and red blood cell counts remained within normal ranges. At 2 years and 6 months after oral eltrombopag initiation, a bone marrow examination was performed to investigate any chromosomal abnormalities caused by eltrombopag. A decrease in megakaryocyte count (13/μL, nucleated cells: 12 × 10^4^/μL) was noted; however, mature megakaryocytes were observed. The donor type was a 99.4% match, and the G-Band showed a normal karyotype. The only adverse reactions observed during eltrombopag treatment were Common Terminology Criteria for Adverse Events Grade 1 hepatic function disorder and diarrhea. There were no adverse reactions requiring treatment interruption or intervention and no ≥ Grade 3 adverse reactions.

## 3. Discussion

This case report showed that in a pediatric patient with PIT, treatment with eltrombopag was effective, and no marked adverse reactions due to eltrombopag were observed.

Several studies have reported on the efficacy of eltrombopag for PIT treatment in adults; however, few studies have reported the same in children. There are six reports on the therapeutic outcomes of eltrombopag use in adult patients with PIT [7,8,9,10,11,12], and in 50–75% of such patients, the platelet counts could be maintained without platelet transfusions using eltrombopag, indicating that there is a certain degree of efficacy in adults. A decrease in the differentiation of megakaryocytes from stem cells has been reported as the pathogenetic mechanism of PIT [13]. Eltrombopag is considered to be effective against PIT because it stimulates megakaryocyte proliferation and differentiation by activating part of the thrombopoietin signaling pathway through specific interactions with the thrombopoietin receptor. There have been some reports on the therapeutic outcomes of eltrombopag use in children with PIT, and many of them support the efficacy and relative safety of eltrombopag [14,15,16]. In our patient, administration of eltrombopag led to a recovery in platelet counts. The patient achieved transfusion independence and a subsequent transplant could be avoided, thereby demonstrating the efficacy of eltrombopag. Although eltrombopag use remains to be fully investigated in children, it may be one of the treatment options for PIT, similar to that in adults.

In our patient, eltrombopag was orally administered for a long time, but no significant adverse events were reported. Chromosomal abnormalities, thromboembolism, and myelofibrosis have been previously reported as significant adverse events associated with eltrombopag. In particular, abnormalities related to chromosome 7 have been reported to occur in approximately 10% of patients and are more likely to occur within 1 month after eltrombopag use. Moreover, eltrombopag has been reported to possibly stimulate leukemia cell division through the thrombopoietin receptor, c-MPL, expressed on megakaryocytic cells [17]. These adverse events should be monitored carefully in pediatric patients with a long survival prognosis, and eltrombopag must be administered with caution. No adverse events have been reported with the long-term use of eltrombopag in children because of the limited experience of use compared with that in adults and the lack of a certain number of prospective follow-ups. In a Phase 2/3, multicenter, randomized, placebo-controlled trial evaluating the efficacy and safety of eltrombopag in children (PETIT, PETIT2) [18,19], long-term use of eltrombopag lasting 24–37 weeks for chronic ITP was reported without major complications; however, chromosomal abnormalities were not evaluated. For our patient, 2 years and 8 months (60 weeks after treatment completion) have passed since eltrombopag treatment initiation, but neither thromboembolism nor myelofibrosis has been observed, and no obvious chromosomal abnormalities have been observed on bone marrow examination. Although the use of eltrombopag should be carefully considered for diseases for which alternative therapies are available, the treatment strategy for PIT is generally blood transfusion therapy and repeated hematopoietic cell transplantation. Because long-term blood transfusion therapy and repeated transplantation greatly impair the quality of life of children with a long survival prognosis, eltrombopag should be considered as a treatment option for PIT. Although our patient is being followed-up for adverse events as a long-term pediatric patient treated for at least 1 year and 10 months consecutively, no significant adverse events have been observed to date. In the future, similar case reports in more pediatric patients and accumulation and analysis of data are needed.

## 4. Conclusions

This case report showed that eltrombopag was effective and safe for treating PIT in a pediatric patient and that it may be one of the preferable treatment options for PIT owing to the limited treatment options currently available.

## Data Availability

Data sharing is not applicable to this article as no datasets were generated or analyzed during the current study.
